# *In situ* assessment of neuroinflammatory cytokines in different stages of ovine natural prion disease

**DOI:** 10.3389/fvets.2024.1404770

**Published:** 2024-10-18

**Authors:** Isabel M. Guijarro, Moisés Garcés, Juan J. Badiola, Marta Monzón

**Affiliations:** Research Centre for Encephalopathies and Transmissible Emerging Diseases. Institute for Health Research Aragón (IIS) – WOAH Reference Laboratory for BSE and Scrapie, University of Zaragoza, Zaragoza, Spain

**Keywords:** cytokine, neuroinflammation, scrapie, prion disease, progress of neurodegenerative disorders, Purkinje cells

## Abstract

**Introduction:**

According to the neuroinflammatory hypothesis, a cytokine-mediated host innate immune response may be involved in the mechanisms that contribute to the process of neurodegeneration. Specifically, regarding prion diseases, some experimental murine models have evidenced an altered profile of inflammatory intermediaries. However, the local inflammatory response has rarely been assessed, and never in tissues from different natural models throughout the progression of neurodegeneration.

**Methods:**

The aim of this study was to use immunohistochemistry (IHC) to *in situ* assess the temporal protein expression of several cytokines in the cerebellum of sheep suffering from various clinical stages of scrapie.

**Results and discussion:**

Clear changes in the expression of most of the assessed markers were observed in the affected sheep compared to the healthy control sheep, and from different stages. In summary, this preliminary IHC study focusing in the Purkinje cell layer changes demonstrate that all cytokines or respective receptors studied (IL-1, IL-1R, IL-2R, IL-6, IL-10R, and TNFαR) except for IFNγR are disease-associated signaling proteins showing an increase or decrease in relation to the progression of clinical disease. In the future, this study will be extended to other inflammatory mediators and brain regions, focusing in particular on the release of these inflammatory mediators by astroglial and microglial populations.

## Introduction

Transmissible Spongiform Encephalopathies (TSEs) or prion diseases are a group of neurodegenerative diseases associated with misfolded prion protein deposits that affect both animals and humans, being along spongiosis, neuronal loss and gliosis, the neuropathological features-hallmarks of this group of diseases. Scrapie is an endemic TSE in many countries worldwide that naturally affects sheep, goats and mouflons. This prion disease has been widely studied and is used in animal models as a prototype of this group of diseases (1). Thus, there is renewed interest in studying this naturally occurring neurological disease in the host species (sheep) to provide reliable information.

Currently, the crucial role of the immune responses in the central nervous system (CNS) of individuals affected with a TSE and other neurodegenerative disorders is being increasingly acknowledged (2–5). In contrast to the theory that described the brain as an ‘immune privileged site’ (6), the neuroinflammation hypothesis postulates that neuroinflammatory mechanisms play a role in the neurodegenerative processes that are associated with brain pathologies such as prion diseases (7–10). Overall, the presence of cytokines has been assessed in several human neurodegenerative diseases, demonstrating that they are produced by different cellular types, including glial cells (11–14). Some studies have proposed that cytokines and other acute phase proteins may contribute to the development of chronic neuropathologies such as Alzheimer’s disease (AD) (15–20).

In TSEs, few studies have focused on the host immune response, probably because it has always been accepted that this group of diseases does not trigger the typical immune response that is observed in other infectious diseases (2). From those studies made in natural model of scrapie, which continue to be a small proportion of all the studies made in prion and prion-like diseases, innate immune responses mediated by dendritic cells, macrophages or microglia have been described during the course of the ovine infection (21, 22). Moreover, an altered profile of inflammatory intermediaries has been specifically shown in some experimental murine models of prion diseases (13, 23–30). However, the local inflammatory response has rarely been assessed (31, 32), and the local inflammatory response has not been previously studied in tissues from different natural models throughout the progression of neurodegeneration. A growing body of evidence indicates that prion protein deposits in a specific anatomical area may act as a stimulus that leads to glial activation and the subsequent cytokine production *in vivo* (31, 32). These signaling proteins can essentially function to activate neural components of the immune response, such as the hypothalamic–pituitary axis, thus serving as regulatory signals between the immune system and the CNS (33). Consequently, the cytokine-mediated host immune response is probably involved in the neuroinflammatory mechanisms that contribute to neurodegeneration (34–36). The actual role of prion protein-induced glial activation and the subsequent cytokine secretion during infection is still incompletely understood. Thus, the involvement of these cytokines in CNS pathology is a growing area of clinical research (34, 37).

Some previous studies about scrapie have focused on the cerebellum because it is a region of the brain that is particularly vulnerable to insults, and abnormalities in this area are usually easy to recognize (38). In addition, the cerebellum is an encephalic area that has been demonstrated to serve as a pseudo reference region to detect neuroinflammation (39). Specifically to scrapie in sheep, our previous studies (40, 41) have shown that Purkinje cells play a relevant role, being at the same time the most damaged and the most protected cellular type in this brain area. These cells are relevant to neurodegenerative progression because constitute the efferent pathway of the cerebellar cortex, playing a pivotal role in the cerebellar circuitry and, as a consequence, their death results in a functional lesion of the cerebellum leading to defects such as ataxia, dysmetria, dysphagia or tremor. Why these cells are the main target of neurodegenerative damage needs to be explained.

This study aimed to ascertain whether inflammatory cytokines could directly affect Purkinje neuron damage. Despite an alteration of these intermediaries had been evidenced in some experimental murine models (24, 27, 30), some authors showed that deficiencies in most of these molecules have little if any influence on CNS prion disease (42–44). As scarce studies have focused on *in situ* tisular expression of these proteins (32, 45) and none of them on a natural model, this is the main novelty and relevance of the present study.

Here, IHC was applied to determine the expression level of IL-1, IL-1R, IL-2R, IL-6, IL-10R, TNFαR and IFNγR in the Purkinje cell layer of the cerebellum from affected sheep throughout the progression of neurodegeneration (at the preclinical, clinical and terminal stages of the disease). Among all the cytokines and their receptors, we decided to focus on these markers because an exhaustive literature review revealed that most authors proposed that these cytokines are significant factors that regulate the pathogenesis of neurodegeneration in experimental models (7–10). To measure some other cytokines of potential interest (e.g., lL-2, IL-10 or IFNγ) was intended but technical problems prevented it.

The objective of this study is to assess the local expression of several cytokines and/or cytokine receptors in this specific affected encephalic area (cerebellum) by studying animals naturally affected by different clinical stages (preclinical, clinical and terminal) of scrapie. To the best of our knowledge, this is the first attempt to evaluate whether the local expression levels of these cytokines and/or receptors vary throughout the natural progression of the disease.

## Materials and methods

This study was approved by the Ethical Committee for Animal Welfare from the University of Zaragoza (Reference number: PI 41/16).

### Samples

Samples corresponded to a total of 31 female sheep ranged from 3 to 9 years old selected from different flocks from *Comunidad Autónoma de Aragón* (Spain) where scrapie was diagnosed by our lab. Different PRNP (prion protein gene) genotypes (ARQ/ARQ, ARQ/VRQ, AHQ/VRQ, and VRQ/VRQ) were included in the study (Table 1). Twenty-three of them corresponded to naturally acquired scrapie sheep at different clinical stages [as previously established in Hernandez et al. (40)]: 7 preclinical (when animal did not present clinical signs but provided positive results for PrP^Sc^ detection by lymph rectal biopsy), 8 clinical (showing nervous symptoms compatible with scrapie) and 8 terminal (when the animal was exhaustively debilitated and prostrated). Eight additional animals were used as healthy controls.

**Table 1:** Full data of sheep analyzed in this study.

Sheep No	*PRNP* genotype	Sex	Age (years)	Group
1	ARQ/ARQ	Female	3	Healthy control
2	ARQ/ARQ	Female	3	Healthy control
3	ARQ/ARQ	Female	3	Healthy control
4	AHQ/VRQ	Female	4	Healthy control
5	ARQ/VRQ	Female	4	Healthy control
6	ARQ/ARQ	Female	3	Healthy control
7	ARQ/ARQ	Female	3	Healthy control
8	ARQ/ARQ	Female	4	Healthy control
9	ARQ/ARQ	Female	4	Preclinical
10	ARQ/ARQ	Female	9	Preclinical
11	ARQ/ARQ	Female	3	Preclinical
12	ARQ/ARQ	Female	6	Preclinical
13	ARQ/ARQ	Female	5	Preclinical
14	ARQ/ARQ	Female	4	Preclinical
15	ARQ/ARQ	Female	8	Preclinical
16	ARQ/VRQ	Female	5	Clinical
17	ARQ/ARQ	Female	5	Clinical
18	ARQ/ARQ	Female	7	Clinical
19	ARQ/ARQ	Female	6	Clinical
20	ARQ/VRQ	Female	9	Clinical
21	ARQ/ARQ	Female	8	Clinical
22	ARQ/ARQ	Female	9	Clinical
23	ARQ/ARQ	Female	6	Clinical
24	ARQ/ARQ	Female	7	Terminal
25	ARQ/ARQ	Female	9	Terminal
26	ARQ/VRQ	Female	5	Terminal
27	VRQ/VRQ	Female	6	Terminal
28	ARQ/ARQ	Female	4	Terminal
29	ARQ/ARQ	Female	6	Terminal
30	ARQ/ARQ	Female	5	Terminal
31	ARQ/ARQ	Female	4	Terminal

After euthanasia with intravenous pentobarbital injection and exsanguination, brains were fixed by immersion in 4% formaldehyde for 48 h and straight paraffin-embedded. Prior to processing, the cerebellum of an hemibrain was sagittally sectioned and processed to be embedded in paraffin.

### Histopathology

Hematoxylin and eosin (H-E) staining was performed on paraffin-embedded 4 μm sections in order to evaluate scrapie lesions such as spongiosis in the Purkinje cell layer of the cerebellum.

### Immunohistochemistry

Immunohistochemical (IHC) protocols were performed in batches using specific monoclonal antibodies to detect prion protein deposition, glial activation and neuroinflammation.

As previously published (46), 98% formic acid immersion for 15 min, proteinase K (4 μg/mL; Roche, Reinach, Switzerland) treatment for 15 min at 37°C and hydrated heating for 20 min preceded the endogenous peroxidase blocking (DAKO, Glostrup, Denmark) for 5 min and incubation with monoclonal antibody L42 (1/500, 30 min RT; DAKO, Glostrup, Denmark) in order to detect prion protein deposition.

For detecting astrogliosis, only endogenous peroxidase blocking (DAKO, Glostrup, Denmark) for 5 min was applied prior slides were incubated with a polyclonal antibody against glial fibrillary acidic protein (GFAP, 1/500, 30 min RT; DAKO, Glostrup, Denmark).

Furthermore, some neuroinflammatory cytokines or cytokine receptors (IL-1, IL-1R, IL-2R, IL-6, IL-10R, TNFαR, and IFNγR) were detected in cerebella from the 31 sheep cited above. After heat treatment (96°C for 10–20 min, depending on the primary antibody; Table 2), endogenous peroxidase blocking (10 min) was applied on all samples. Then, slices were incubated with the respective primary antibody following Manufacturer’s instructions (overnight at 4°C; further information in Table 2): Anti-IL1RN (Sigma, Sweden); monoclonal mouse anti-Human CD30 (clone Ber-H2, DAKO, Denmark); CD25 monoclonal antibody, IL-10RA monoclonal antibody, IFNγR1 polyclonal antibody, IL-1 alpha polyclonal antibody, IL-6 monoclonal antibody (ThermoFisher, Denmark).

**Table 2:** Details about the primary antibodies used for each marker assessed including specific pre-treatment for their detection by immunohistochemistry in paraffin embedded samples.

Antibody	Antigen	Type	Original target species	Dilution	Specific pretreatment	Source
IL-1 alpha	IL-1	Polyclonal	Human	1:100	Formic acid 15 min 96°C 20 min	ThermoFisher
Anti-IL-1RN	IL-1R	Polyclonal	Human	1:100	Formic acid 15 min 96°C 20 min	Sigma
IL-2R.1	IL-2R	Monoclonal	Human, Mouse	1:1.000	96°C 10 min	ThermoFisher
8H12	IL-6	Monoclonal	Human	1:20	Formic acid 15 min 96°C 20 min	ThermoFisher
OTI1D10	IL-10R	Monoclonal	Human	1:250	Formic acid 15 min 96°C 20 min	ThermoFisher
CD30,clone Ber-H2	TNFαR	Monoclonal	Human	Ready to use	96°C 15 min	Dako
IFNGR1	IFNϒR	Polyclonal	Human	1:200	96°C 20 min	ThermoFisher

For all primary antibodies cited, secondary antibody was added depending on the primary antibody used: Envision anti-mouse or anti-rabbit (DAKO, Denmark) for 30 min and DAB visualization system was performed (10 min). Slides were counterstained with hematoxylin, dehydrated and mounted with DPX mounting media (DAKO, Denmark).

Immunolabeling magnitude and intensity was specifically examined in the 31 samples for each marker for cytokines or receptors by using light microscopy focusing on Purkinje cell layer. It was subjectively scored from 0 to 4; i.e.: 0 = absence of labeling; 4 = intense brown labeling in Purkinje cell layer (it comprises Purkinje cells and some other surrounding components, as glial cells). Two observers independently evaluated blinded samples by counting positive cells in 5 microscopic fields in cerebellum. The density and the extent of the labeled deposits in each sample examined was assessed as previously published by the group (47, 48).

Specificity was validated by replacing the primary antibody with an unmatched isotype (Supplementary Figure 1).

### Statistical analysis

The normal distribution of values was analyzed with the Kolmogorov–Smirnov test. Statistical analysis of the intensity data between groups was performed using one-way analysis of variance (ANOVA) followed by Bonferroni post-hoc test using the SPSS software (SPSS Statistics for Windows, Version 17.0).

Graphics were performed using the GraphPad software (GraphPad Prism, Version 6.01). All data were expressed as mean values ± SEM. Differences between groups were considered statistically significant at *p* < 0.05 (*) and *p* < 0.01 (**).

## Results

### Neuropathology at different stages of disease

**Figure 1:**
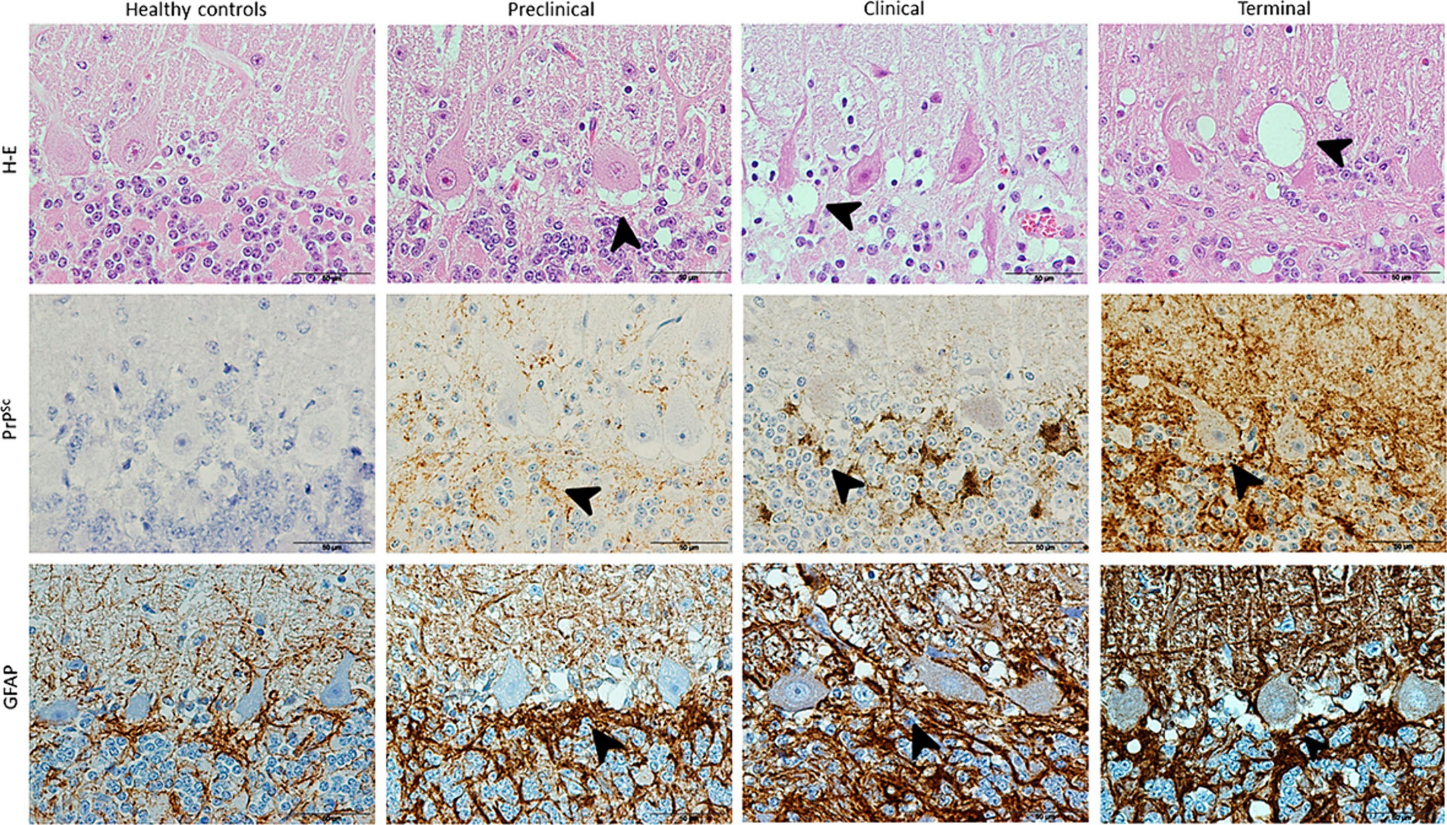
Images illustrating neuropathological hallmarks of prion diseases (arrowheads: vacuolation, PrP^Sc^ deposition and glial activation) in scrapie-affected sheep at different clinical stages. Note that H-E technique reveals vacuolation in Purkinje cell layer in a higher extent as scrapie progresses and IHC shows large PrP^Sc^ deposits and associated glial activation (brown; hematoxylin counterstaining, blue) in all diseased sheep, reaching maximum in terminal stage.

The results provided by neuropathological assessment in all animals studied is showed in Figure 1. As evidenced here, spongiosis, PrP^Sc^ deposition and astrogliosis increased in severity in scrapie brains and also as disease progress. Those lesions were absent in controls for the two first markers.

Spongiosis around Purkinje cells increased with scrapie progression, initially spreading to granular and later also to molecular layer. The degeneration of these neurons was evident from clinical until death in terminal stage.

PrP^sc^ deposit and GFAP immunostaining was shown to follow the same order than described for spongiosis, being first more evident their accumulation in granular layer (clinical stage) and finally extending to molecular layer too.

### Cytokine kinetics in the Purkinje cell layer

**Figure 2:**
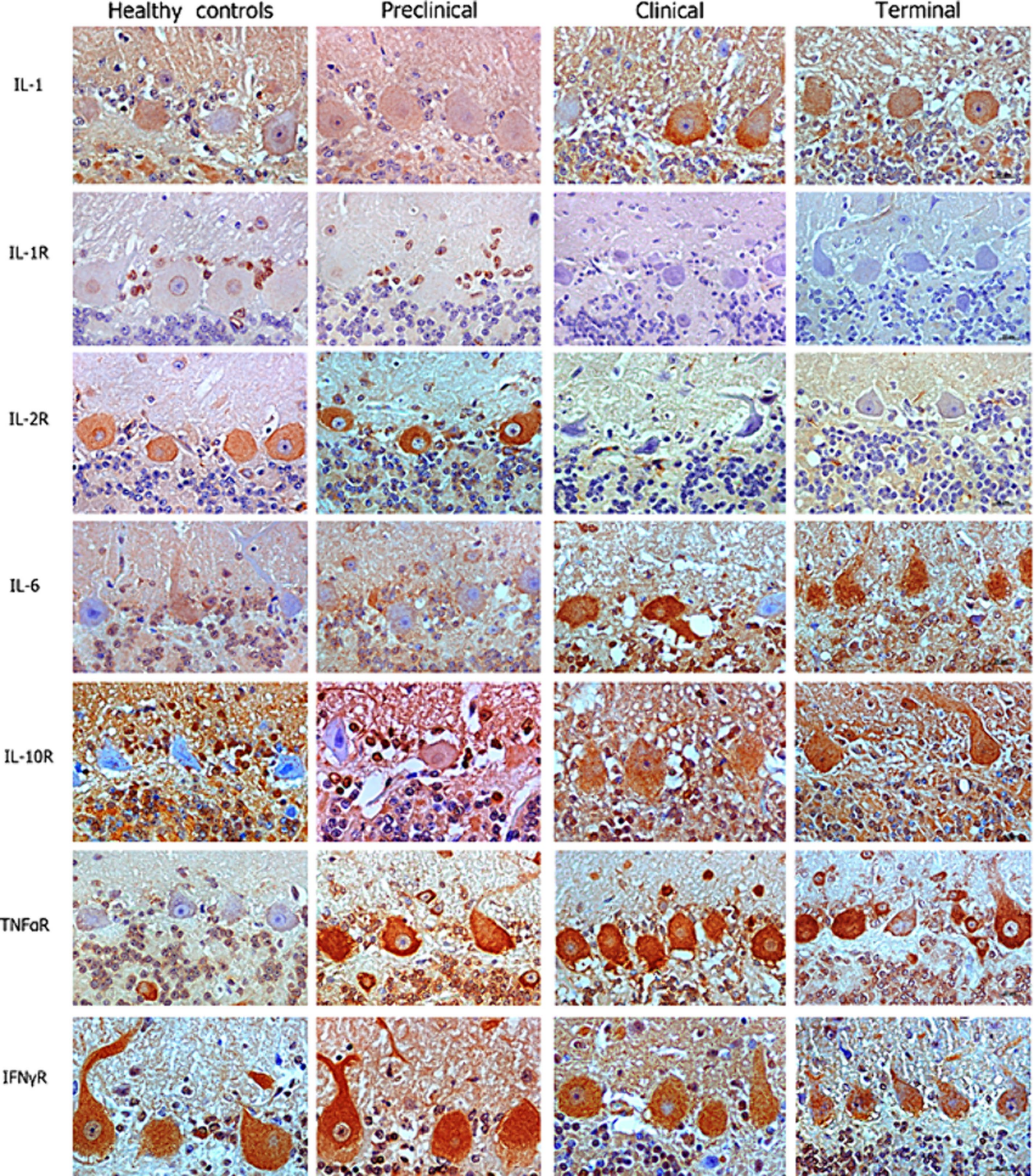
Images corresponding to Purkinje cell layer from cerebella corresponding to healthy, preclinical, clinical, and terminally scrapie affected sheep (*columns*) immunostained with antibodies (*rows*) against different neuroinflammatory cytokines (brown; hematoxylin counterstaining, blue).

**Figure 3:**
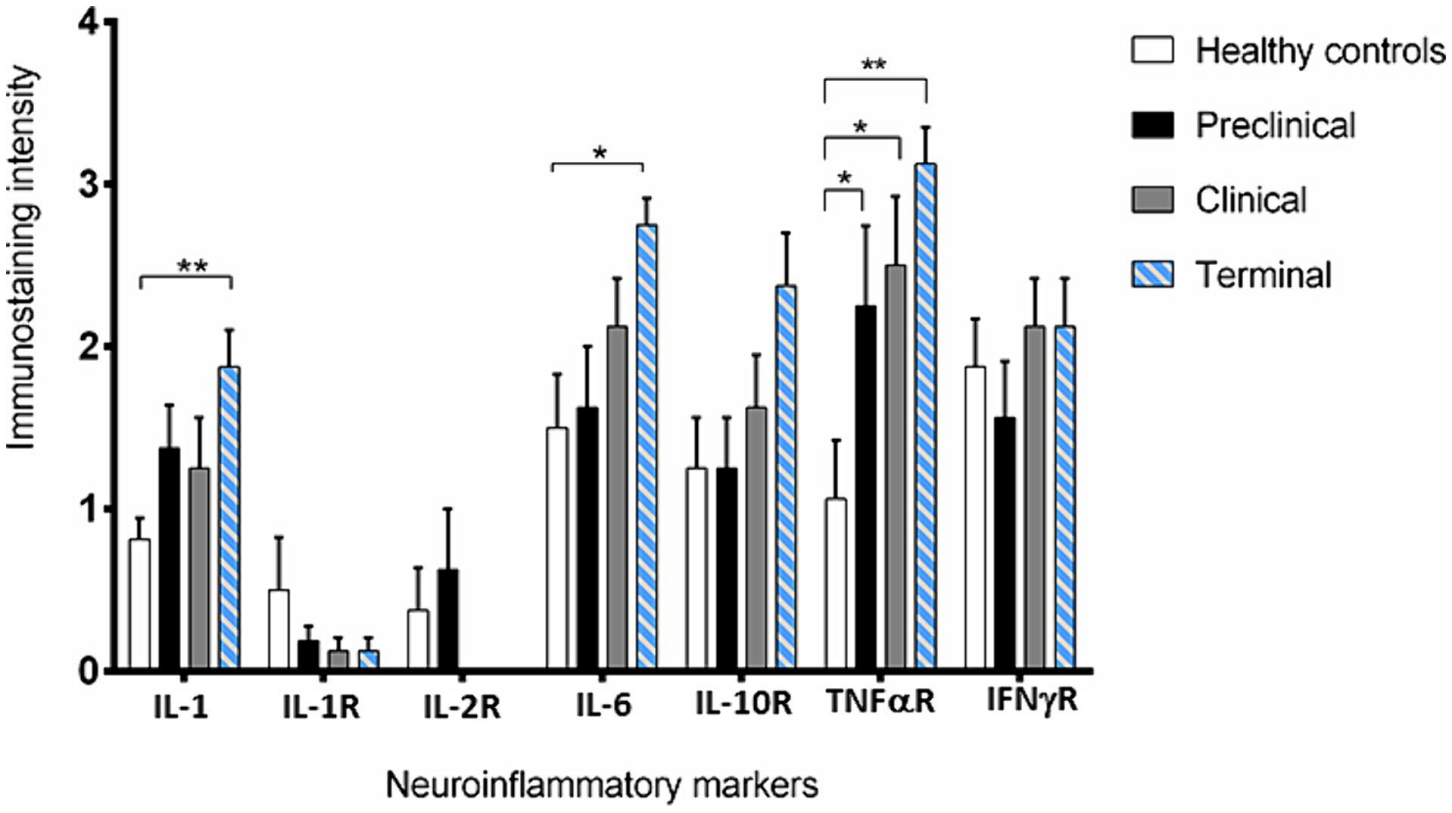
Intensity values for each neuroinflammatory marker scored in the 31 samples studied by immunohistochemistry. Data are expressed as mean ± SEM. Differences between groups are considered statistically significant at *p < 0.05 and **p < 0.01. By Bonferroni post-hoc, significant differences were found between immunostaining intensity at terminal stage of scrapie and healthy controls for IL-1 (**), IL-6 (*) and TNF-αR (**). In addition, they were also found for TNF-αR between control and the clinical and terminal stages of the disease (*).

Our IHC results revealed that Purkinje cells expressed high levels of different neuroinflammatory markers in healthy and diseased brains. But overall, an increased abundance of immunolabeling of Purkinje cells was shown in the scrapie -affected sheep (in all stages) than in the healthy control sheep (Figure 2). The most outstanding results concerning each cytokine or receptor assessed on the basis of images and statistical study shown in Figures 2, 3, respectively, are described below.

IL-1 and IL-6 profiles were analogous. A basal level of expression was observed in the Purkinje cells from the healthy control sheep, being the expression of these pro-inflammatory cytokines significantly increased with scrapie progression. The expression of these cytokines reached maximum intensity in these neurons when the animals were at the terminal stage of the disease. In fact, statistically significant differences were found in the expression of IL-1 (**p < 0.01) and IL-6 (*p < 0.05) between the healthy control sheep and the sheep in the terminal stage of scrapie.

IL-10R expression was quite similar in healthy and preclinical sheep. However, although not reaching significant differences, the immunostaining intensity noticeably increased in clinical and terminal stages, specially inside these neurons.

Concerning TNFαR, a progressive increase was observed in the sheep from the preclinical stage of scrapie, showing statistically significant differences when compared any disease stage affected to the healthy sheep (*p < 0.05 in preclinical and clinical, and **p < 0.01 in terminal stages).

By contrary, a substantial decrease in IL-1R was observed in the preclinical compared with healthy sheep. Even more evident decrease was observed when clinical and terminal sheep were compared, because scarce immunostaining was observed. Moreover, it was mainly observed in small cells around the Purkinje cells but not in the Purkinje cells themselves.

IL-2R expression was slightly increased (not statistically significance) in the sheep in the preclinical stage compared to the healthy control sheep, but the immunostaining intensity became absent in clinical stages.

Finally, no clear changes were observed in IFNγR expression over the course of scrapie.

**Figure 4:**
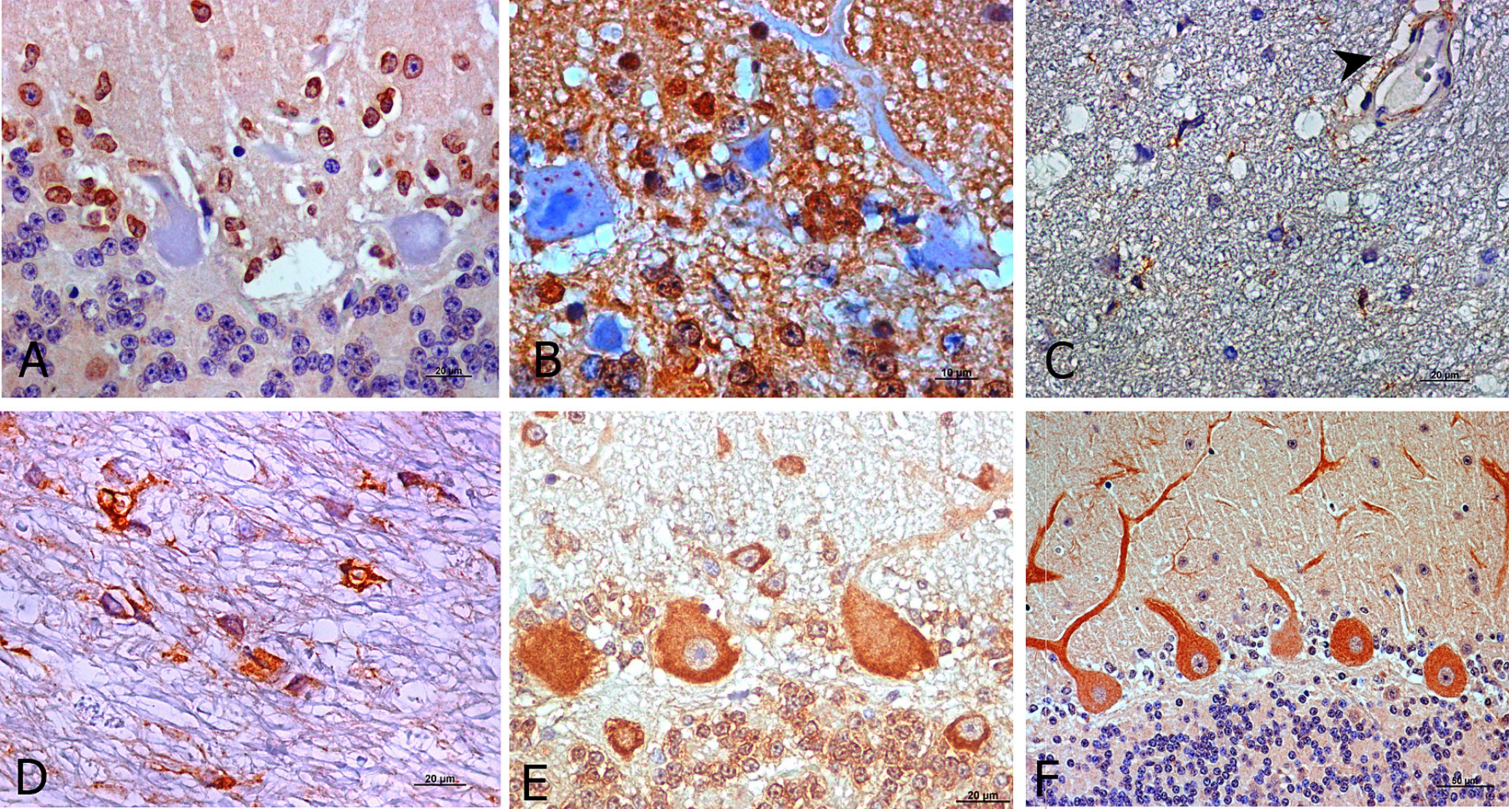
Outstanding morphological findings provided by light microscopic examination after IHC for detection of neuroinflammatory cytokines (brown; hematoxylin counterstaining, blue). Several immunopositive cells for IL-1R at preclinical stage exhibited staining likely corresponding to astrocytes **(A)**. IL-10R was expressed as intra-cytoplasmic spots in Purkinje cells from healthy control sheep **(B)**. In addition, IL-2R immunostaining during clinical **(C)** and terminal stages **(D)** was frequently expressed by pleomorphic cells but in other than Purkinje cells and it was also present on some blood vessels with thick walls (**C**, arrowhead). TNFαR was also expressed in other cerebellar layers (granular and molecular) than in Purkinje cells in all stages (**E**, preclinical). For IFNγR, the immunostaining pattern appeared more uniform in Purkinje cell dendritic spines, mostly in healthy controls as well as preclinical stage of scrapie (**F**, preclinical).

Regarding cell-associated immunostaining patterns, Purkinje cells were the most immunoreactive cells. However, mainly for IL-1R, cells with an astrocyte-like morphology that surround Purkinje cells exhibited specific immunostaining in healthy and preclinical scrapie -affected animals (Figure 4A). Moreover, the soma of the Purkinje cells did not exhibit immunoreactivity with this specific antibody.

When IL-10R was assessed, immunolabeling of both, the Purkinje cells and cells surrounding them, was observed. The soma of Purkinje cells from healthy control sheep exhibited scarce intracytoplasmic spots in healthy and in early diseased animals (Figure 4B). Meanwhile, this pattern transformed into a pattern that was similar to that described for the other markers in the subsequent disease stages.

As stated, IL-2R immunostaining in the healthy and preclinical animals was mainly observed in the Purkinje cells, while during the clinical and terminal stages, it practically disappeared from these neurons. However, it was then observed in an easily identifiable pleomorphic cell type (Figures 4C,D) mainly located in the white matter, as shown in Figure 4D. This marker was also present in blood vessels with thick walls (Figure 4C).

In the case of TNFαR, immunolabeling was initially expressed by the Purkinje cells in the preclinical stage. But as scrapie progressed, it was also expressed by other cells in other cerebellar layers (Figure 4E).

The IFNγR immunostaining pattern appeared to be more uniform in the dendritic spines and soma of Purkinje cells (Figure 4F).

## Discussion

This study provides the inflammatory kinetics of a few innate-immune system related protein markers in the Purkinje cells of cerebellum from sheep with natural scrapie. Our results showed evident changes in the expression of most of the assessed markers for cytokines/receptors in the prion -affected compared to the healthy control sheep.

Previous reports did not observe an association between the upregulation of any pro-inflammatory cytokines and the course of disease by means of IHC (49), probably because, as introduced, natural models were not used nor were cytokines assessed *in situ*. However, the technique applied here shows differences in the expression of almost all of the assessed cytokines in the scrapie -affected sheep compared to the healthy control sheep, supporting the indisputable role of cytokines in the pathogenesis of TSE. Particularly, compared to the healthy control sheep, the clinical and terminal sheep expressed significantly increased levels of IL-1 and IL-6, while in contrast, the clinical and terminal sheep expressed decreased levels of IL-1R and IL-2R. Moreover, IL-10R and TNFαR expression was substantially increased from the preclinical stage onwards, and the expression of TNFαR in all the stages of scrapie was significantly higher compared to that in the healthy sheep.

As mentioned before, we considered it of interest to determine cytokine expression throughout the progression of neurodegeneration in a natural model. Previous studies of cytokines in models of prion diseases have not done so. Most of the knowledge about neuroinflammation in prion diseases has come from studies using transgenic mouse models. Our study contributes to the growing research on the complex relationship of natural prion diseases with cytokines. However, the upregulation of these mediators requires further research to confirm or exclude its possible detrimental role in chronic neurodegeneration. The fact that cytokines play a key role in neuroinflammation and neurodegeneration has opened new areas of scientific investigation.

There are several studies of prion diseases in which the expression of pro-inflammatory cytokines has been described (24, 25, 31), often in the context of the onset of different clinical stages. However, the profile of cytokines described here differs from previous results, as an increase in some cytokines was detected even at the preclinical stage of scrapie. The differences might be because none of these previous studies focused on the progression of neurodegeneration, and they did not use natural models.

Here, it is shown that IL-1 is highly expressed in ovine scrapie, mainly at the clinical and terminal stages. This interleukin is one of the main pro-inflammatory cytokines and, consequently, its overexpression its linked with neuroinflammation. Likewise, Cunningham et al. (26) demonstrated an overexpression of this cytokine in a mouse model of prion disease. In fact, the early overexpression of IL-1 has been suggested to be a candidate mechanism that contributes to the development of murine scrapie (27), given its relationship with neuroinflammation. This suggestion is consistent with our results in sheep. Actually, the presence of an impaired cytokine response in mice orally infected with scrapie, which mimics the natural route of entry (50), has been described. Similarly, IL-1 gene expression has been associated with susceptibility to scrapie in sheep, and differences in the expression of this cytokine have been shown in the cerebellum but not in the spleen (9). By IHC, we confirmed here the differences in IL-1 expression in ovine cerebellar tissue. Similar to studies of AD in humans (16, 51, 52), we observed that IL-1 is overexpressed in the preclinical animals with scrapie compared to the healthy controls.

To date, there are studies indicating that IL-6 can have both neurotrophic and neurotoxic effects on CNS neurons (53). In addition, IL-6 has been reported to exert trophic effects on glial cells, particularly oligodendrocytes (54). In this study, we found that the expression of this cytokine was significantly increased in the Purkinje cells in the clinical stage of scrapie and reached the maximum expression in the terminal stage. Therefore, this observation is in agreement with previous reports, in which the overexpression of IL-6 was demonstrated to produce neurological disease in mice, activating astrocytes and microglia (55). Moreover, IL-6 was shown to activate astrocytes *in vitro* (56). In our results, a basal level of this marker was observed in the healthy controls. Indeed, it has been previously described it is constitutively expressed in discrete regions of the CNS not only during illness but also under normal physiological conditions (57).

As explained above, natural models that provide reliable conclusions about neurodegenerative disorders are rarely available. Nevertheless, it has been reported that the anti-inflammatory cytokine IL-10 is neuroprotective concerning prion disease (29) and traumatic brain injury (58), respectively. It has been suggested that this cytokine not only plays a neuroprotective role by acting on IL-10R present on the surrounding neurons but also by increasing the secretion of TGF-1β by astrocytes, which is known to play an important role on the interaction between microglia and astroglia. That could explain the increase of the expression of the IL-10R throughout the scrapie progression observed in the present experiment, and it remarks the importance that the glial cells have in the development of prion and prion-like diseases.

TNFαR staining was consistently observed from the preclinical stage of disease, demonstrating that cytokine immunoreactivity was present before the appearance of clinical signs; this outcome agrees with the report by Williams et al. (48). However, from the preclinical stage onwards, a significant increase in the immunostained Purkinje cells was detected here, and it lasted until the terminal stage of the disease. The meaning of this overexpression, and whether it corresponds to either neuroprotective or neurotoxic roles as suggested by other authors (42, 59), is still unknown. However, since TNFα not only causes the production of cytokines but also plays an important role in the extrinsic apoptosis pathway, changes in either TNFα or TNFαR could be linked with changes in this process of cellular death. More scientific research on this topic should be done in order to clarify the link existing between apoptosis and neurodegeneration.

In the present study in sheep, several IL-1R immunopositive cells in the Purkinje cell layer of the preclinical animals exhibited a morphology consistent with that of astrocytes, which was consistent with previous studies using murine models (25, 27, 31, 45). Nevertheless, one remarkable finding was that neither IL-1R nor IL-2R were increased from the preclinical stage onwards. In fact, there was a progressive decrease in both of these markers in our study. Taken together, these data indicate that, similar to IL-1, a role for IL-2 receptors in TSE neuropathology cannot be excluded. Additionally, previous studies had reported that some cytokine receptors are localized in the granular layer but not in the Purkinje cell layer in rats (60). In contrast, other authors have found that immunostaining for such receptors in the granular layer was low and that this immunostaining was higher in the Purkinje cells in mice (61). This latter distribution is similar to that observed in our study; the cytokine receptors were more highly expressed by the Purkinje cells than by other cells in the sheep cerebellum.

On the basis of results provided in the present study regarding IFNγR, this cytokine is suggested not to play a significant role in natural ovine scrapie.

As cited, some problems arose regarding background in IHC applied here, as previously described by Parker and Smith (62). A possible explanation could be that the recommended PLP fixation and low-temperature paraffin embedding, described as ideal protocols for the IHC detection of cytokines in mouse tissues (63), were not followed in the present study. But most likely, the commercially available antibodies are not as specific for detecting the target epitopes in sheep. Another limitation of the study consisted of the range of age between control and affected animals. However, natural infection studies depend on sample availability.

This preliminary study shows an inflammatory profile in the cerebellum of sheep naturally affected by scrapie at different stages of the disease. Provided that our results suggest a relevant immunological component in the progression of natural neurodegeneration, in the future, this study will be extended to include other inflammatory proteins and brain regions to provide a global profile of the neuroinflammatory process in a natural model of this group of prion diseases, potentially extrapolable to other neurodegenerative disorders. Both quantitative and molecular studies to identify the specific cell types expressing these receptors have been initiated with the objective of elucidating the consistent differences among the distinct disease stages. The observed differences in terms of expression levels confirm that a complex network of cytokines is involved in the pathogenesis of natural scrapie. But moreover, these immunostaining patterns of cytokines would potentially serve as neuroinflammatory markers throughout natural scrapie progression and also as therapeutic target. For instance, Gabay (64) proposed blocking IL-6 as a possible treatment against chronic inflammatory diseases.

By identifying the cell type that expresses these inflammatory mediators, paying particular attention to astroglial and microglial populations as the main components of the host immune response in prion diseases, reliable conclusions about the neurodegeneration process can be also drawn. Undoubtedly, the characterization of cytokines in glial cells will enable us to gain information about the bidirectional communication between the immune and nervous systems. Understanding the actions of cytokines in scrapie and other prion and prion-like diseases could lead to novel therapeutic strategies.

## Data Availability

The original contributions presented in the study are included in the article. Further inquiries can be directed to the corresponding author.

## Supplementary material

SUPPLEMENTARY FIGURE S1 Validation of specificity of the primary antibodies for target in ovine species was assessed by replacing them with an unmatched isotype. Images show absence of unspecific immunosignal in both control and Scrapie animals for all antibodies tested.
