# Supplementary figures and images for: In situ assessment of neuroinflammatory cytokines in different stages of ovine natural prion disease
Source: Front Vet Sci. 2024 Oct 18;11:1404770. doi: 10.3389/fvets.2024.1404770 (PMC11528339; doi:10.3389/fvets.2024.1404770)

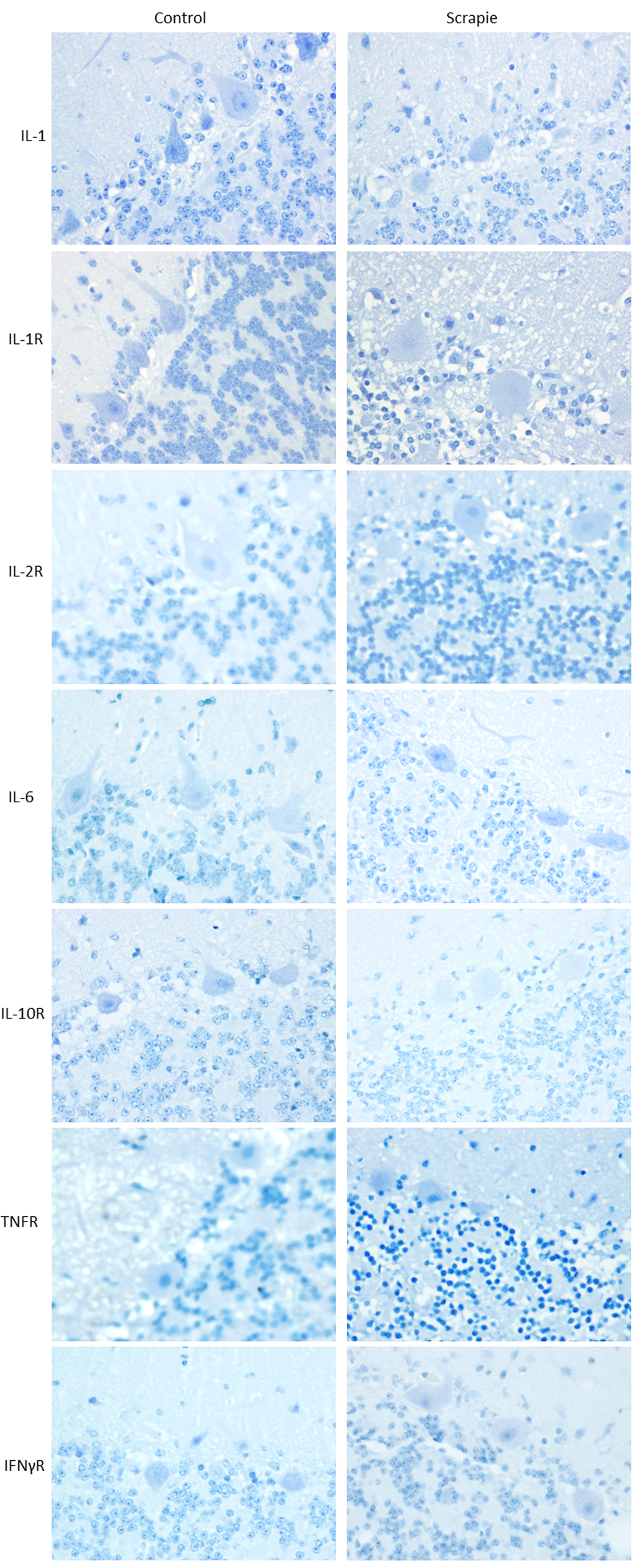

Supplement: SUPPLEMENTARY FIGURE S1 — Validation of specificity of the primary antibodies for target in ovine species was assessed by replacing them with an unmatched isotype. Images show absence of unspecific immunosignal in both control and Scrapie animals for all antibodies tested. [file Image_1.TIF]
